# Facilitators and barriers to integration of noncommunicable diseases with HIV care at primary health care in Ethiopia: a qualitative analysis using CFIR

**DOI:** 10.3389/fpubh.2023.1247121

**Published:** 2023-12-08

**Authors:** Abebe Sorsa Badacho, Ozayr Haroon Mahomed

**Affiliations:** ^1^School Public Health, Wolaita Sodo University, Sodo, Ethiopia; ^2^School of Nursing and Public Health, Public Health Medicine Discipline, Durban, South Africa; ^3^Health Economics and HIV and AIDS Research Division (HEARD), University of KwaZulu-Natal, Durban, South Africa; ^4^Dasman Diabetes Institute, Kuwait City, Kuwait

**Keywords:** integration, models, CFIR, PHC, PLWH, NCD, HIV

## Abstract

**Background:**

The rise in non-communicable diseases (NCD), such as hypertension and diabetes among people living with human immunodeficiency virus (PLWH), has increased the demand for integrated care due to multiple chronic care needs. However, there is a dearth of evidence on contextual factors implementing integrated hypertension and diabetes care with HIV care. This study aimed to identify facilitators and barriers that could affect the integration of hypertension and diabetes with HIV care at primary health care in Ethiopia.

**Methods:**

Five primary health facilities from five districts of the Wolaita zone of South Ethiopia were included in the qualitative study. Fifteen key informant interviews were conducted with healthcare providers and managers from the zonal, district, and facility levels from October to November 2022. Data collection and analysis were guided by a consolidated framework of implementation research (CFIR).

**Results:**

Ten CFIR constructs were found to influence the integration. Perceived benefit of integration to patients, healthcare providers, and organization; perceived possibilities of integration implementation; availability of NCD guidelines and strategies; a supportive policy of decentralization and integration; perceived leaders and healthcare provider commitment were found to be facilitators. Perceived increased cost, insufficient attention to NCD care needs, inadequate number of trained professionals, inadequate equipment and apparatus such as blood pressure measurement, glucometers, strips, and NCD drugs, inadequate allocation of budget and weak health financing system and poor culture of data capturing and reporting were identified as barriers to integration.

**Conclusion:**

It is important to address contextual barriers through innovative implementation science solutions to address multiple chronic care needs of PLWH by implementing integrated hypertension and diabetes with HIV care in primary healthcare. Training and task shifting, pairing experienced professionals, and strengthening the health care financing system to implement evidence-based integration of hypertension and diabetes are recommended.

## Background

The Sub-Saharan African (SSA) population faces a double burden of infectious and noncommunicable diseases (NCDs) (1, 2). NCD-related morbidity and death are predicted to rise significantly in SSA (2). In SSA countries, cardiovascular diseases (CVDs) are the leading cause of NCD burden and pose an increasing challenge for health systems, which have primarily focused on struggling with infectious diseases and maternal, neonatal, and child health issues (2).

The successful expansion of antiretroviral therapy (ART) has increased the life expectancy of people living with HIV (PLWH), and HIV has become a chronic disease (3, 4). HIV infection and ART exposure are risk factors for developing NCDs such as CVD and diabetes (3–6). PLWH are at a higher risk of developing NCDs than HIV-negative persons because some HIV medicines increase blood glucose levels and lead to diabetes (7, 8). PLWH comorbid with NCDs show poor immunologic restoration and are at high risk for immunologic failure (3).

The World Health Assembly adopted the Global NCD Action Plan in 2013 (9, 10), which aimed to reduce the preventable and avoidable burden of morbidity, mortality and disability due to NCDs through multisectoral collaboration and cooperation at national, regional, and global levels (9, 10). The Global NCD Action Plan focused on a global monitoring framework with six strategic objectives and nine voluntary global targets, including the first global target of a 25% relative reduction in overall mortality from the four major NCDs (CVD, cancers, diabetes, and chronic respiratory diseases) by 2025 (9) and further reinforced by the 2030 agenda of one-third (33%) reduction of premature mortality due to NCDs as universal health coverage (9, 11).

The World Health Organization (WHO) (12) has recommended proven, cost-effective interventions as a package of essential NCD interventions (13), expanded to all primary healthcare (PHCs) and fully integrated and supported by appropriate referrals and regular follow-up (14). There is growing evidence of the efficacy and cost-efficiency of integrated chronic-disease management models based on person-centered care (2). An integrated people-centered approach is recommended by strengthening health systems to provide universal, sustainable, and quality services (12), which enables people to receive the care they need when they need it in user-friendly ways (15, 16).

Despite effective, proven NCD interventions, their implementation is inadequate in SSA countries (9, 13). In sub-Saharan Africa, the coverage of NCD services remains poor, and evidence of adherence and retention in care is limited (16, 17). In Ethiopia, the PHC health system has low NCD service readiness, a shortage of NCD-trained workforce, equipment, and medicines, and insufficient funding for NCD (18).

Due to the lack of integrated HIV and NCD care in Ethiopia, PLWHs do not receive routine hypertension and diabetes screening for diagnosis at the same ART clinic (18). The absence of integrated HIV-NCD services increases the burden on PLWH by increasing transportation costs, medication prices, and missed productive days (19).

Ethiopia has adopted policies, strategies, and NCD guidelines, such as the establishment of a National Strategy Action Plan (NSAP) focused on expanding PHC service packages (20) and guidelines for NCD intervention (21). However, progress in providing an integrated NCD service has been minimal, particularly at the PHC level (22–24). Ethiopia’s PHC system mainly involves acute care delivery and infectious disease prevention (24). In recent years, cervical cancer screening has been initiated for those women receiving ART in Ethiopia. However, the implementation of screening women living with HIV for cervical cancer is weak (25). The sustained, coordinated provision of NCD/HIV services for PLWH is essential to address individuals with HIV and NCDs and help attain ART’s aim of prolonging life (26).

Before adopting HIV and NCD integration, it is essential to understand the facilitators and barriers that may influence the integration of hypertension and diabetes with HIV care, as well as contextual factors and providers’ viewpoints on the integration model (14). This study aimed to explore service providers’ and managers’ perspectives on integrating NCD (hypertension and diabetes) in the PHC context for HIV treatment in Ethiopia.

## Methods

### Study area and setting

This study was conducted in the Wolaita zone of Southern Ethiopia, with an estimated total population of 5,385,782. Wolaita Zone of south Ethiopia is one of the second leading with 4,900 people living with HIV, followed by the Gofa zone with 9,700 south Ethiopia region in 2022. The zone has one teaching referral hospital, two private general hospitals, and 75 PHC Units (including 7 Primary hospitals and 68 health centers and 358 health posts).

### Study design

A descriptive qualitative approach.

### Selection of study participants

Five PHCs which provide ART services were purposively selected from each from five districts of the Wolaita zone were included in the study. Fifteen key informants purposefully selected representing one senior management, one HIV program coordinator from Zonal level, five PHC directors, three NCDs, and five HIV focal persons purposively selected were included in the in-depth key informant interview (KI). Participants were approached for in-person, in-depth interviews.

### Data collection and procedures

Data was collected from October to November 2022. The Principal Investigator (PI), who speaks the local language, Amharic, fluently, conducted in-depth interviews using an interview guide adapted to CFIR 1.0 domains and constructs (27). In-depth interviews were conducted at offices in the health facility, and participants’ privacy and confidentiality were maintained. The interviews were voice-recorded, and the Amharic language was used. None of them refused or dropped out of the study. Only the participant, principal investigator, and research assistant were present during the interviews. No repeated interviews were conducted. The interview times ranged from 50 to 80 min. Voice-recorded in-depth interviews were transcribed, verifying the transcribed interviews.

### Data analysis

When the official language Amharic, was used, the data were transcribed and translated back to English by an Amharic language-speaking translator and checked for accuracy and completeness. The transcribed data were rechecked using the original data to confirm consistency. Multiple reviews of transcripts and voice records were conducted to familiarize the data. NVivo software was used for data processing and analysis. All transcripts were imported into NVivo software for qualitative data analysis and assisted with data organization. Deductive analyses were conducted to contextualize the research findings (28). The thematic framework analysis using CFIR domains and constructs guided the analysis (27).

### Trustworthiness of the study

The principal investigator and research assistant were fluent speakers of the participants’ native language and culture and familiar with the study community. In this study, credibility was ensured by purposive sampling in selecting the study participants who were eligible for participation. These participants were key to our research questions. The research team for this study had prior experience with qualitative research and an adequate academic background.

## Results

Fifteen key informant interviews were conducted; eleven (73%) were male, four (27%) were female, six (40%) participants had a master’s degree, seven (47%) with a bachelor’s degree, and two with medical doctorate degrees. Two of the responders were members of senior management. One-third of the participants (33%) were ART focal person, three (20%) were NCD focal persons, and five (33%) were PHC directors. One-third (33%) and 40% of participants, respectively, had more than 15 years and 11 to 15 years of PHC work experience (Table 1).

**Table 1:** Socio-demographics KI participated in in-depth interviews to identify facilitators and barriers to integrating NCD with HIV care for PLWH at PHC of South Ethiopia, November 2022.

Characteristics		Frequency (N)	Percent (%)
Age	25–34	5	33
	35–44	7	47
	>45	3	20
Sex	Male	11	73
	Female	4	27
Year of experience	5–10 years	4	27
	11–15 years	6	40
	> 15 years	5	33
Education status	BSc degree	7	47
	Master’s degree	6	40
	MD degree	2	13
Current Responsibility	ART Focal person	5	33
	NCD Focal person	3	20
	PHCU Director	5	33
	HIV Program Coordinator	1	7
	Senior Manager	1	7

### Theoretical framework used to present the findings

The facilitators and barriers are presented using the Consolidated Framework for Implementation Research (CFIR).

**Figure 1:**
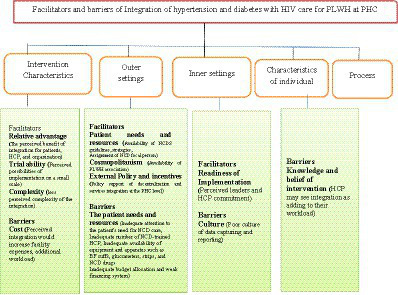
The CFIR, with five principal domains and 10 of the 39 underlying constructs, was identified as the most significant potential facilitator and barrier to integration.

The CFIR domain of intervention characteristics (relative advantage of intervention, trialability, complexity), outer setting domain (patients’ needs and resources, cosmopolitanism, external policy and incentives), and inner setting domain (readiness for implementation) were found to be facilitators. The intervention characteristics (cost), outer setting domain (patients’ needs and resources), inner setting domain (culture), and characteristics of the individual domain (knowledge and belief in intervention) were identified as barriers to integration (Figure 1).

## Facilitators for integration

### CFIR domain 1: the intervention characteristics

#### The complexity of the integration at PHC

Most of the participants indicated that integrating hypertension and diabetes services with HIV care sounded feasible and that screening of hypertension and diabetes among PLWH at PHC would not be complex. However, they emphasized the importance of training ART providers in NCD service provision, task shifting, pairing experienced HCPs, and availing manuals.

*“I do not think integrating became complex… The intervention of screening might not be complex. If the training and manuals are available, the screening of hypertension and diabetes might not be complex. However, diagnosis and management of diabetes might need resources.” (Female, ART focal)*

*“It is not a complex screening diagnosis of hypertension and diabetes because the reason is for screening professionals trained during their study, and some professionals get updated training on NCD it is possible and not complex.” (Male, ART focal)*

*“For a better change, it needs starting what you have. … For example, at the health centre level, we have professionals trained in NCD and shifting them is possible. And are pairing those trained and experienced with others to implement screening diagnosis and treatment of hypertension and diabetes at the ART clinic for PLWH. It is possible starting with what you have; for example, in OPD, there are BP cuffs and a glucometer machine at the laboratory. It is possible by sharing the machines and starting screening and diagnosis of hypertension and diabetes for PLWH.” (Male, ART focal)*

#### The relative advantage

All participants believed integrating hypertension and diabetes with HIV care would benefit patients, HCPs, and the organization.

##### The perceived relative benefit for patients

All participants mentioned that integrated provision could be used for early detection of hypertension and diabetes that could help patients to know their hypertension or diabetes status early as possible before the onset of complications due to these conditions. In addition, participants perceived that integration could improve the treatment adherence for ART treatment and hypertension and diabetes control, improving patient outcomes and quality of life.

*"Integration has many benefits in improving patient outcomes; For PLWH using HIV care at ART providing NCD services integrated with HIV care, the first thing to remember is that most of the time, opportunistic infection and related factors harm PLWH more than HIV infection. Integrating hypertension and diabetes care at ART clinics may help improve HIV care outcomes. By preventing OI and allowing PLWH to adhere to ART, PLWH can have life expectancies comparable to other HIV-negative people. Without NCD screening and prevention, this OI deteriorates PLWH immunity, decreases the quality of life, and affects HIV care outcomes." (Female, Leader)*

Another ART focal added that, in her experience, many PLWH members die without knowing their status because of a lack of hypertension and diabetes services at the ART clinic. She emphasized that integrating could help early detect these conditions and save premature deaths.

*"Integration of hypertension and diabetes in HIV care and treatment has many benefits in prolonging and improving quality of life. Patients with HIV, when viral load is suppressed, and there is good adherence to HIV treatment, can live a similar life to others without HIV; however, due to NCD, patients are dying, and their quality of life is compromised…Hypertension is a silent killer, and routine screening is essential for early detection and prevention activities before the onset of complications due to the disease. It would help save the patients' lives. PLWH members on ART are dying due to NCD. I have experienced many PLWH members in HIV care who died without knowing their hypertension and diabetes. The patients die without getting treatment due to a lack of screening for hypertension and diabetes here, the patient dies. If the services are available could have saved their lives." (Female, ART Focal)*

Most participants perceived that integration would minimize patients’ concerns and fear of discrimination and stigma when receiving services from multiple places and visiting different providers. Participants also noted that offering NCD care and treatment at ART clinics for PLWH; could maintain privacy, reduce multiple visits for different services, and help patients access comprehensive services in one location, saving patients time and money on several trips for different services.

*“When PLWH members receive hypertension and diabetes care with other patients within OPD, they feel afraid about the identification of their HIV status by others, leading them to stigma and discrimination. They are expected to wait a long time; the PLWH members, when going to OPD, they are expected to have another medical card. If ART clinics could provide NCD care and treatment for them [PLWH], In that case, they could quickly get all services in one place, which might help them to access hypertension and diabetes care would help to improve their health. It might save their time, keep their privacy and confidentiality, reduce visiting many providers and places, and reduce exposure to stigma and discrimination.” (Male, ART Focal)*

##### The perceived relative benefit to HCP

Participants believed combining hypertension and diabetes care with HIV care at PHCs will benefit HCPs by enhancing knowledge and skills through task sharing and capacity building in NCD service provision. Additionally, integrated HIV and NCD could help HCP gain confidence when treating their patients and would help HCP deliver comprehensive care for PLWH.

*"From the perspective of the HCP, there could be task sharing and building the HCP's capacity on NCD service delivery. Because the ART clinic lacks integrated hypertension and diabetes care, HCP only provides HIV care and faces challenges when PLWH presents with diabetes and hypertension. Due to a lack of NCD services, HCP face challenges. When HCPs are linked with ART clinics and can provide treatment for patients, they can gain confidence in treating hypertension and diabetes which would help them develop their knowledge and skills to give NCD care.” (Male, Leader)*

Another ART Focal added that the integrated provision of services would help them to provide comprehensive patient care, which helps to boost HCP satisfaction by providing needed services.

*“If services are integrated…professionals could gain experience in both NCD and HIV care provision. HCP could provide comprehensive patient care if NCD and HIV services are integrated. … Professionals could provide both services in one place, making patients trust them[HCP] because patients could get all services in that room, improving their health. Integrated services provision could help boost HCP satisfaction, and HCP could feel proud to provide needed services for PLWH. if integrated services could be provided at the ART clinic, I would be happy and satisfied caring for PLWH; most of the time, I feel guilty because I am not providing all needed care to my clients due to a lack of comprehensive care for PLWH primarily NCD services are not provided here, even patient present with signs.” (Female, ART Focal)*

##### The perceived benefit to the organization

All participants stated integration would improve the organization’s performance and service delivery efficiency by enhancing screening, early detection, and linkage to chronic care, which might improve PLWH health outcomes and reduce NCD-related deaths.

*“If ART patients could be screened for hypertension and diabetes at the ART clinic, the organization’s performance would improve. It will improve PHC’s execution of NCD initiatives. ……. providing NCD services at the ART clinic could enhance patient adherence to ART care and patient quality of life. ……Another advantage of integrating is that it will connect HIV and NCD service delivery, both critical programs. ……. This project will connect two critical programs that touch many people: HIV and NCDs. In my opinion, this program is a significant effort that can potentially influence long-term NCD care provision at a wide community level in the future.” (Male, HIV Coordinator)*

#### Trialability of the integration

Participants were optimistic that integrating hypertension and diabetes care with HIV care in PHC facilities could be successful. Various stakeholders would support this intervention, which would be successful and scaled up in a large setup that would benefit patients.

*"As a pilot program, if we begin integrating hypertension and diabetes care with ART for PLWH, leaders of the zonal health department, the regional health bureau, and other stakeholders can support integration implementation. Furthermore, the Ministry of Health may be able to expand this program to other parts of the country. As an organization and as zonal health department administration, we will support and ensure the success of the integration of hypertension and diabetes with the ART clinic. I feel it will be successfully implemented. I'm hoping that the integration will benefit the clients. We see it as a good idea that could benefit the clients." (Male, Leader)*

## CFIR domain 2: outer setting

### Facilitators of patient needs and resources

#### Availability of NCD guidelines and assigned NCD focal persons

The availability of national NCD guidelines, strategies, and targets and NCD focal persons assigned at PHC level and participants noted that at least one physician had been assigned to ART clinics of PHC who can provide NCD treatments to PLWH and could be facilitators for integrating hypertension and diabetes within HIV care.

*“Nationally, there are NCD guidelines, strategies, and targets, as well as zonal and woreda-level NCD and NTD focal persons assigned. These could be integration facilitators. The national NCD guidelines can be utilized as a facilitator for guiding…We have at least one person trained in NCD at the woreda level. we have trained NCD focal assigned at facilities; …. At least one physician is available at each of the 20 ART providing facilities. Others HCP may consult physicians while providing services. Physicians are assigned to ART clinics and, if they received NCD training, can provide NCD treatments to the PLWH…As previously stated, we have NCD and NTD focal persons at the zonal, woreda, and health facility levels…All facility levels have NCD/NTD focal persons. NCD focal can interact and consult with ART focal and other leaders before beginning this initiative will be implemented successfully” (Male, HIV coordinator)*

#### PLWH associations

Participants remarked that the PLWH associations used leveraging to increase ART adherence and cervical cancer screening for PLWH, which could be a good opportunity for integration.

*“The PLWH association is utilized to send a message to patients who have stopped taking ART medicines, and helped an ART medication adherence. They are supportive, and most of the clients that came for cervical cancer screening did; they were aware of the presence of services from their association. Associations will aid in the screening for hypertension and diabetes care and treatment, as well as provide a conduit for message transmission. They have a monthly gathering to listen to each other and discuss their problems…It could be an excellent opportunity to screen for hypertension and diabetes, among other things.” (Male, HIV coordinator)*

#### Supportive policy of decentralization and integration of services

Participants stated that the current health policy supports the implementation of NCD interventions, encourages service integration, and focuses on the decentralization and integration of services at PHC. In addition participants noted that some facilities have started piloting the integration of different services activities at PHC.

*“Regarding the Policy, there is no challenge to providing integrated hypertension and diabetes care with ART clinics because, currently, the government policy focuses on decentralization and integration of the services at PHC. For example, integration helps to reduce unmet needs. In this regard, there are initiatives for service integration at the pilot level. …. some integration activities are at the pilot level…I do not think regarding Policy there is a problem. Currently, Policy encourages and supports the integrated provision of services, especially at the PHC level” (Male, Leader)*

## CFIR domain 3: inner setting

### Perceived leadership commitment

The participants believed that leaders and HCP would be committed to implementing NCD and HIV care integrated at the PHC. They hoped that integration could generate a lesson of evidence from the implementation, which can be used to scale at other regions and the national level.

*“Regarding leadership commitment, I am a program coordinator. We are committed, and there is no problem with leadership commitment. We will be ready with other stakeholders to implement integrated NCD and HIV care at the PHC level. I can tell you that the management and team leaders would be committed to the implementation. I am a zonal HIV program coordinator willing to support the implementation. I would like to express my commitment. I hope we will come up with a result in a short period of time if we start the implementation; it is imperative. I hope a good lesson of evidence could be generated if we implement the integration of hypertension and diabetes within the ART clinic for PLWH. It can be a good lesson for other regions and at the national level.” (Male, HIV coordinator)*

Additionally, a senior manager noted that leaders would be committed to integration. However, it needs to involve all leaders from top to bottom in awareness-creation activities to gain ownership of the program and for sustainability.

*“Regarding leadership, awareness creation activities should be focused on starting from top to bottom before integration when the new program implementation requires aware the leaders from the top to health extension workers. Health centre directors, hospital managers, and all leadership levels should be involved and oriented to work responsibly, and accountability can make to gain ownership and sustainability of the program.” (Male, Leader)*

## Barriers to integration

### CFIR domain 1: the intervention characteristics

#### Cost of the intervention

Participants expressed concern that integration would increase facility expenses. However, given the benefits to patients, there should be a means to cover the expenditures. Participants pointed out that ART services are provided for a free fee for PLWH, and previously opportunistic infection (OI) drugs were free of charge even though they are currently not provided for free. Participants fear integrating hypertension and diabetes with HIV care because PLWH could expect the free provision of hypertension and diabetes care with HIV care. Also, participants noted their fear that integrating NCD care with HIV care would increase HCP workload and that could add management costs for the provision of hypertension and diabetes services.

*"In terms of HCPs, it may add additional workload to ART services. It will increase management costs because ART services are free, and patients would demand free-of-fee hypertension and diabetes services, which will incur facility costs…This may increase the HCP workload and add to management costs. …as I previously stated, PLWH receives HIV care free of fee, ART drugs are free of charge, and stakeholders previously provided free OI drugs. For example, various OI drugs, such as amoxicillin for adults, syrup for children, and cloxacillin, are provided free of charge. However, these OI drugs are not currently available for free. As a result, providing this hypertension and diabetes care might put a burden on the facilities. Health centres with minimal revenue generating will face financial challenges when implementing integration." (Male, Leader)*

### Domain 2: outer setting

#### Barriers related to patient needs and resources

##### Lack of attention to the PLWH need for NCD care

All participants expressed concern about the lack of attention to screening NCDs such as hypertension and diabetes, particularly among PLWH. Screening at the ART clinic focused on TB and OI with signs and symptoms, leaving hypertension and diabetes undetected.

*“Hypertension and diabetes go untreated, …The most important measure to take to avoid OI. OI is harming our clients. …. OI kills our patients, but there is little attention to identifying and treating OI, such as hypertension and diabetes. We only treat OI with signs and symptoms. …if we could accurately diagnose diseases like diabetes and hypertension, early detection could save a life. Hypertension and diabetes received low attention…we only focused on tuberculosis screening and treatment. NCDs, on the other hand, should be given priority and could be screened and diagnosed early. However, there was low focus on NCD prevention activities.” (Male, ART Focal)*

##### Inadequate number of NCD-trained professionals

Participants noted that a shortage of HCPs trained in NCD at PHC facilities could hinder the integration of hypertension and diabetes with HIV care since NCD services are provided at secondary level care in Ethiopia.

*"There is a problem with early screening for hypertension and diabetes from service points. The first issue is a shortage of NCD-trained HCP; ……These are the most serious issues with early screening and detection. Yes, at the level of the health center… The first is a shortage of NCD-trained healthcare workers, which may hinder integration. As far as NCDs are concerned, NCD care is provided at the hospital level." (Female, Leader)*

##### Inadequate equipment and apparatus

Participants mentioned the main challenge that could hinder integrating hypertension and diabetes care with HIV for PLWH at PHC are lack of equipment such as BP cuffs, glucometers, strips, and drugs and supplies. In addition, the health facilities’ capacity to purchase these supplies due to raising the prices of the material in the current market is a challenge. These issues should be solved before starting the implementation of the integration of NCD and HIV care at the PHC level.

*“The primary barrier to combining hypertension and diabetes care with HIV services for PLWH could be a lack of equipment and supplies, such as blood pressure cuffs, glucometers, strips, and other supplies and medicines. The difficulty of the health facilities' purchasing capacity to get supplies, medical equipment, and pharmaceuticals due to rising supply and medical equipment prices.” (Male, Leader)*

The ART focal added that due to a lack of machines and equipment, they used to refer patients to private clinics, but patients leave without getting treatment.

*“For the routine screening for hypertension and diabetes for PLWH, we have a problem with screening due shortage of machines and equipment for screening hypertension and diabetes. Sometimes machines are not functional in the facility, and we mainly send the patients to private clinics to be checked for their blood pressure and blood sugar level because we lack machines to check blood pressure and blood sugar levels. When we refer our clients to private clinics, they leave not treated and went home without getting services because they have economic problems paying for services from private clinics.” (Female, ART Focal)*

##### Inadequate budget allocation and weak financing system

Participants stated that their facilities provide most of the services, such as maternal care services, family planning, tuberculosis diagnosis and treatment free of charge. A lack of reimbursement or fee waiver system to cover the services cost of exempted services poses a financial burden on the facilities, jeopardizing the provision of services at the PHC. Also, participants noted insufficient healthcare funds and support for NCD service provision.

*“Many government health facilities offer free services to clients, and there is no reimbursement system, posing a financial burden on the facilities. The healthcare finance of the institutions is inadequate and failing due to free services such as maternal care, tuberculosis, family planning, vaccination so on…This could challenge facilities to provide free hypertension and diabetes care to PLWH. Another issue is that stakeholder support for NCD service provision is lacking. The Federal Ministry of Health provides ART medications and other opportunistic infection treatments to PLWH with the support of donors. It would be preferable to coordinate hypertension and diabetic medications included in ART centrally, and the opportunistic infection therapy drug list, and making them available to PLWH would assist clients in improving their health outcomes.” (Male, Leader)*

Participants stressed that there is a problem at the market and supply agencies supplying medical equipment at the national level. Participants noted that the problem of budget shortage, budget distribution, and budget utilization at national and local levels could hinder the integration of hypertension and diabetes care with HIV care at PHC facilities.

*“There is a problem at market and supply agencies, with supplies and even facilities to purchase. Supply problem in supplying medical equipment and supplies at a national level. There is a problem at the local level, woreda level lack of budget, budget distribution, and budget utilization and timely budget provision.” (Male, Leader)*

### Domain 3: inner setting

#### Poor data capturing and reporting

Participants reported that there is a good culture of sharing resources and understanding each other when providing care. However, a poor data-capturing culture was identified could be a barrier to integration.

*“As an organization, we have good working culture; we share resources; for example, even for providing HIV care, we have a shortage of gloves. I can use gloves from MCH, Laboratory, and different departments and use resources by shifting. We have a culture of sharing resources from different departments and understanding each other.” (Male, ART focal)*

*“Poor data capturing culture at the moment makes things difficult, but we can still address this issue by regularly monitoring and evaluating.” (Male, HIV coordinator)*

### Domain 4: characteristics of individuals

Participants emphasized that while patients can view the integration as having a good impact, professionals may see it as adding to their workload. Prior to beginning integration, it is crucial to have a discussion with HCPs and stakeholders about the program’s significance.

*“Considering integration from the viewpoint of experts. Professionals may feel that their burden has increased as a result of the services integration. There could be some discomfort as well as resistance. Concerning the patients, they will be optimistic and hopefully delighted to receive the care. One PLWH came in to be examined for more than just hypertension and diabetes; they also expected to be screened for TB and cervical cancer. Receiving comprehensive care and undergoing tests for TB, mental health, diabetes, hypertension, and other conditions may take some time for the patients.” (Female, Leader)*

#### Suggested strategies for integration of NCD with HIV care at PHC

The three models proposed for NCD integration within PHC were presented to HCP for suggestions. The first option was the integration of NCDs (hypertension and diabetes) and HIV care at the NCD clinic for all patients; the second option was the integration of NCDs (hypertension and diabetes) and HIV care at the ART clinic for all patients, including PLWH. The third option was the integration of NCDs (hypertension and diabetes) and HIV care for only PLWH at the ART clinic. All participants suggested that integrating hypertension and diabetes care with HIV care provided for PLWH at the PHC would be feasible. They would prefer that hypertension and diabetes care be included in the ART package and provided as routine care with ART services. ART providers added their fear that mixing other HIV-negative patients for hypertension and diabetes care at the ART clinic would affect HIV care services. HCP raised the issue that PLWHs fear stigma and discrimination when receiving services with other patients who are not using ART and leave receiving the services. Also, ART providers pointed out their experience when referring PLWH to the NCD clinic for hypertension and diabetes service; patients leave receiving NCD services.

*“I would suggest that hypertension and diabetes screening and follow-up services should be included in the ART package, and the reporting of hypertension and diabetes also be integrated with medical cards with ART cards for follow-up. I strongly suggest availing of NCD services at the ART clinic combined with HIV care and treatment and giving due attention to hypertension and diabetes screening as the other opportunistic infections like TB.” (Male, ART Focal)*

The other ART focal person added that PLWHs do not want to receive services from other HCPs than ART providers.

“*They would be happy with the diagnosis provided at ART clinics; they fear stigma and discrimination when using it with others. They don’t want to get services with other non-HIV patients in the same room because they fear their HIV status is disclosed to others, leading to stigmatization. Even they don’t want to receive care from other than ART providers. Because they fear stigma and discrimination when their HIV status could be identified, they even want to take drugs from pharmacies and HCPs who don’t know them. I strongly suggest adding hypertension and diabetes care to the ART package and components of ART services for the sustained provision of hypertension and diabetes care for PLWH.” (Male, ART focal)*

The HIV program coordinator emphasized that integrating hypertension and diabetes with HIV care at ART clinics for PLWH would be possible based on their experience of HIV prevention activities.

*“The option of arranging NCD screening, diagnosis, and treatment follow-up of hypertension and diabetes for PLWH at the ART clinic would be the best. Because we have lived experience regarding services rendered to ART users, we understand very much about services provided for ART users. I strongly recommend screening hypertension and diabetes among ART users at the ART clinic. It could be better-availing services for the ART users at the ART clinic rather than them queuing with other patients at the OPD level” (Male, HIV coordinator)*

The fairness in NCD service provision was emphasized and noted by participants that PLWHs are more prone to NCDs, including hypertension and diabetes, and have a greater need for NCD services than other groups of people.

*“…it is a concern of fairness that those who need the services more should access the services; due to HIV infection, PLWH is more prone to hypertension and diabetes, which makes special needs for the PLWH.” (Female, Leader)*

## Discussion

This qualitative study explored potential facilitators and barriers to integrating hypertension and diabetes with HIV care for PLWH at the PHC from providers’ perspectives. We took a theory-informed approach using CFIR domains and constructs to identify barriers and facilitators that may be addressed to strengthen and sustain the diagnosis of hypertension and diabetes at PHC. CFIR has been designed to be flexible, allowing researchers to adapt the framework to the intervention design, underlying variables, and reviewed settings, making connecting these findings to subsequent implementation strategies simpler.

Our study showed that HCP and managers believed integrating hypertension and diabetes with HIV care would benefit patients, HCPs, and the organization. This relates to the CFIR construct of the relative advantage of integration identified as a potential integration facilitator from the inner setting domain of CFIR. Participants perceived that an integrated provision could be used for the early detection of hypertension and diabetes before the onset of complications due to these conditions. Our study finding consistent with a study conducted in Tanzania reported that integration benefited patients in knowing their hypertension and diabetes status, and earlier detection helped to prevent further risks and complications (29). NCDs diagnosed earlier have a greater opportunity for reducing NCD-associated complications (30). Additionally, our study finding was in line with what was reported elsewhere in Tanzania (31), Malawi (32) and Kenya (33).

HCP perceived integration could improve the treatment adherence for ART, hypertension and diabetes control, improving patient outcomes and quality of life. Our finding was consistent with the Tanzania study, which showed that integrating NCD with HIV care improved patient adherence to treatment, lifestyle modification, and good viral load suppression (29). Integration of chronic care in Uganda increased adherence to HIV care and improved blood pressure control by more than threefold (34). Implementing an Integrated Chronic Diseases Model (ICDM) in South Africa indicated a higher probability of controlling blood pressure and improved CD4 count (35). Integrating care for HIV, diabetes and hypertension achieved high retention levels in care for people living with diabetes or hypertension in Africa (36).

Participants perceived that integration would minimize patients’ fear of discrimination and stigma when receiving services from multiple places and visiting different providers. Our study finding was consistent with the studies in South Africa; the ICDM model conferred an advantage on PLWH because of the reduced stigma due to the non-segregation of patients managed for chronic disease in the same clinic (35, 37). In contrast, Tanzania’s study showed the integration of hypertension and diabetes and HIV services; patients perceived integrated services to have infringed upon their privacy (29); Due to clinic facilities being accessible to other patients and a single room serviced all of the professionals, who would sit there and confer with patients (29).

Participants also noted that offering NCD care and treatment at ART clinics for PLWH members could reduce multiple visits for different services and could help patients access to comprehensive services in one location, saving patients time and money on several trips for different services. Our study was consistent with other studies; integrated care enhanced access to care, as reported in Malawi, South Africa, Swaziland, and Kenya (38). Tanzania’s study reported that integration saved time and the costs of visiting multiple clinics and enabled patients to engage in economic activities (29). Uganda study reported that the integration of hypertension and HIV care reduced redundant visits to facilities (34).

Participants believed that integration could help HCP gain confidence when treating their patients and help HCP deliver comprehensive care for PLWH. This finding was consistent with the Tanzania study; integration helped build patient trust and ensured a friendly and conducive environment (29). In our study, Participants noted that the availability of NCD guidelines, strategies, and targets could facilitate integration. Our study finding was consistent with the Tanzania Dar es Salaam study, which reported the availability of national guidelines on managing NCDs among PLWH enablers of NCD integration (39).

Participants believed that leaders and HCP would be committed to implementing NCD and HIV care integrated at the PHC. They hoped that integration could generate a lesson of evidence from the implementation, which could be used to scale up other regions and the national level. A study conducted by Tanzania Dar es Salaam indicated that positive attitudes among PLWH and HCP toward integrating NCD services within HIV care noted as facilitators of integration (39).

In our study, Participants noted inadequate attention to NCD prevention activities, particularly for PLWH, which could be a barrier to integration. Similarly, qualitative research in Ethiopia explored that NCDs received little attention at all levels. This was noted as a key reason why NCD programs are poorly implemented at the PHC level (22). NCD service delivery is generally disconnected from community care and implemented as a vertical program concentrated in hospital facilities in Ethiopia (22, 40). For PLWH care, focus limited to HIV care was reported as a barrier to integrating NCDs (41). Our finding was consistent with a qualitative study of Tanzania, Dar es Salaam study; the lack of priority given to the recognition and treatment of NCD care by HCP and patients were identified as a barrier to the integration of NCD for PLWH (39).

As potential barriers, our study identified inadequate human resources such as a shortage of HCPs trained in NCD at PHC facilities, which could hinder the integration of hypertension and diabetes with HIV care at PHC. Our study finding was consistent with a prior qualitative study in Ethiopia that showed a shortage of qualified HCP barriers in delivering services for chronic NCDs (22); it seriously hinders offering assistance and counseling (42). A qualitative study conducted at Tanzania Dar es Salaam; reported that lack of regular training on NCD was found to be a challenge in integrating NCD with HIV care for PLWH (39). Another study from Tanzania reported that a scarcity of trained providers to deliver integrated care was a challenge in implementing integration (29). Shortage of well-trained health professionals and inadequate knowledge and skill in NCD case management are challenges for the health workforce during decentralization, leading to compromised case management and inconsistent risk assessment (24). The implementation of ICDM in South Africa; staff shortages were reported as barriers to providing quality ICDM care (33).

Inadequate equipment, such as BP cuffs, glucometers, strips, supplies, and NCD drugs, could hinder integration. A prior qualitative study in Ethiopia reported that the availability of equipment needed for detecting or treating NCDs in health facilities indicated significant deficiencies (22, 42). Previous studies noted that limited PHC facilities with functional BP apparatus, glucometers and other equipment impacted NCD care implementation (22). Our study finding was consistent with Tanzania’s studies; occasional unavailability of random blood glucose (29), the absence and inconsistency of supplies and the lack of medicine were reported as potential barriers to integrated services for PLWH comorbid with NCDs (36). The implementation of ICDM in South Africa; malfunctioning blood pressure machines and staff shortages were reported as barriers to providing quality ICDM care (33).

Participants raised the issue of insufficient healthcare funds and support for NCD service provision could be barriers to integration. Even though maternal and child health services, HIV and TB are provided with a fee-wavering approach in Ethiopia, there are high out-of-pocket payments (OOP) related to services (43). Household OOP healthcare expenditure accounts for one-third of total healthcare expenditure and barriers to access to care, especially for NCD services (44). Limited government financing for PHC facilities results in high catastrophic household OOP expenditures connected to NCD treatment because of a lack of financial protection; scarce resources for NCDs are disproportionately allocated to tertiary care facilities (22). Insufficient healthcare funds and lack of support for NCD service provision are barriers to integration, leading to high OOP, limited government funding, and enormous disparities in seeking healthcare (22, 44). A Tanzania study reported that the biggest challenge was financing NCD treatment costs for PLWH-integrated HIV and NCD (39). Studies found that PLWH, due to financial barriers, challenged access to hypertension and diabetes care (41).

## Limitations of the study

The current study was conducted in one region and purposefully selected participants. Due to the varying health system performance profiles, the findings may not be generalized to other regional or sub-national entities.

Although participants strongly recommended integrating hypertension and diabetes into their HIV care clinics for PLWH at PHC is feasible with suggested solutions to the identified barriers, we appreciate that this decision is theoretical and needs testing and evaluation to make further recommendations.

The ART package given to PLWH at the HIV care clinic at the time of data collection did not contain hypertension or diabetes. As a result, it was unable to evaluate whether it would be possible to diagnose diabetes and/or hypertension with HIV in PLWH.

This study used a qualitative approach that provided an in-depth understanding of contexts that may facilitate or hinder integrating hypertension and diabetes services into HIV care at PHCs in Ethiopia. We recognize that the results of this study may only be applied to other contexts similar to the studied populations.

## Conclusion

Integrating hypertension and diabetes care into the HIV care clinics at PHC for PLWH would be feasible.

The critical considerations for successfully integration need to address contextual barriers and enhance facilitators for integration. Appropriate implementation strategies are suggested to address barriers to integration, for instance, to offset rising costs and make integrated services more accessible through health insurance enrollment. Integrated NCD with HIV chronic care as collocated services, a well-trained workforce, and clinic infrastructure will likely be crucial to address multiple chronic care needs of PLWH (34). Training ART providers on NCD provision and task shifting to provide NCDs, motivation and pairing experienced HCP, and strengthening the health care financing system may be harnessed to implement evidence-based integration of hypertension and diabetes with HIV care at PHC.

## Data availability statement

The original contributions presented in the study are included in the article/supplementary material, further inquiries can be directed to the corresponding author.

## Ethics statement

The studies involving humans were approved by Ethical approval was obtained from the UKZN Biomedical Research Ethics Committee (BREC) with protocol reference number BREC/00003857/2022. The studies were conducted in accordance with the local legislation and institutional requirements. The participants provided their written informed consent to participate in this study.

## Author contributions

ASB and OHM: conception and design of the study and data analysis. ASB conducted interviews. All the authors have read and approved this manuscript.
